# A study of the inter- and intra-operator variability on selected echocardiographic measurements in dogs

**DOI:** 10.1007/s11259-023-10154-6

**Published:** 2023-06-23

**Authors:** Sara Ghilardi, Danitza Pradelli, Rita Rizzi, Michele Polli, Mara Bagardi, Roberto A. Santilli, Paola G. Brambilla, Claudio M. Bussadori

**Affiliations:** 1 Department of Veterinary Medicine and Animal Sciences – DIVAS, University of Milan, Lodi, LO 26900 Italy; 2 Department of Cardiology, Clinica Veterinaria Gran Sasso, Milan, MI 20131 Italy; 3 Anicura Clinica Veterinaria Malpensa, Samarate, VA 21017 Italy; 4 Department of Clinical Sciences, Cornell University, Ithaca, NY 14853 USA

**Keywords:** Echocardiography, Reliability, Reproducibility, Repeatability, Gold standard

## Abstract

Standard transthoracic echocardiography is considered the non-invasive gold standard for the diagnosis of most cardiac diseases. Defining reproducibility, repeatability, and reliability of this exam is imperative to reduce errors in clinical evaluations. The present study aimed at: (1) evaluating the reproducibility and repeatability of 15 echocardiographic parameters in dogs by analyzing measurements obtained from several operators with different levels of experience and comparing them to the ones obtained from two board-certified operators (gold standards - GSs); (2) assessing whether different formative paths have an influence on the variability of the echocardiographic measurements. Fifty-one operators have been included in this study, along with two diplomates of the European College of Veterinary Internal Medicine - Cardiology. Ten dogs were enrolled, 5 Golden Retrievers and 5 Cavalier King Charles Spaniels. Echocardiographic examination was performed on each dog by one GS and several operators on the same day. Results show the highest deviation from the GS and a poor inter-operator reproducibility for the M-mode measurements of the interventricular septum and the left ventricular free wall. Differently, M-mode-obtained internal diameters of the left ventricle in systole and diastole, and measurements of the aortic annulus and root show moderate to excellent intra- and inter-operator reliability and a good concordance with the GSs, demonstrating that all the operators correctly assess left ventricular systolic function and dilation, and evaluate the aortic valve. Furthermore, a specialist clinical activity, more than the acquired theoretical knowledge, affects the reliability of the echocardiographic examination by reducing the difference from the GS’ measurements.

**Supplementary Information:**

The online version contains supplementary material available at 10.1007/s11259-023-10154-6.

## Introduction

The standard transthoracic echocardiography is a widely spread technique used in veterinary medicine, and it is considered the non-invasive gold standard for the diagnosis of most cardiac diseases (Thomas et al. 1993; Chetboul and Tissier 2012). The examination is often practiced not only for the long-term follow-up and the drug efficacy trials, but even to assess day-to-day changes in hospitalized patients (Chetboul et al. 2003). The interpretation of the echocardiographic examination depends on qualitative and quantitative assessments of the heart, for this reason, intra- and inter-operators’ variability must be primarily assessed in order to understand if variations of the obtained measurements are to be attributed to disease progression. Indeed, small changes may be due to observer-dependent factors, also (Dukes-McEwan et al. 2002). Defining reproducibility, repeatability, and reliability of the echocardiographic examination is imperative to reduce errors in clinical evaluations. Reproducibility is defined as the variation of the same measurement made on a subject under changing conditions (such as different operators); repeatability is the variation in repeat measurements made on the same subject under identical conditions; lastly, reliability is the magnitude of error between repeated measurements (Bunting et al. 2019; Popovic and Thomas 2017). In the past few years, a growing interest has emerged on this topic. In 2002, Dukes MacEwan et al. assessed the reproducibility of 65 echocardiographic parameters on six Boxer dogs and found that M-mode measurements are the most reliable ones (Dukes-McEwan et al. 2002). In the dog, several other echocardiographic measurements have been analyzed: parameters used to evaluate pulmonary artery pressure (Abbott and Gentile-Solomon 2017), linear and volumetric measurements of the left atrium (Hsue and Visser 2020), the evaluation of mitral valve prolapse (Pedersen et al. 1996), measurements of left atrial and left ventricular size and function (Visser et al. 2019), and measurements on the right heart (Gentile-Solomon and Abbott 2016; Morita et al. 2017). In the cat, a few studies reported the coefficient of variation of several echocardiographic parameters, in sedated cats (Moise et al. 1986) as well as in awake cats (Chetboul et al. 2003; Simpson et al. 2007; Van Hoek et al. 2018). In particular, the study conducted by Chetboul et al. (2003) focused also on the variability of the exam when measurements were made by observers with different levels of experience (Chetboul et al. 2003). Finally, even in other species such as horses (Schwarzwald et al. 2007, 2009; Slack et al. 2012; Worsman et al. 2022), calves (Lecoq et al. 2018) and goats (Leroux et al. 2020), different authors have tried to assess reliability of the transthoracic echocardiography. In all these studies, only a few operators were engaged (one to four) (Moise et al. 1986; Pedersen et al. 1996; Dukes-McEwan et al. 2002; Chetboul et al. 2003; Schwarzwald et al. 2007, 2009; Simpson et al. 2007; Gentile-Solomon and Abbott 2016; Abbott and Gentile-Solomon 2017; Morita et al. 2017; Lecoq et al. 2018; Van Hoek et al. 2018; Visser et al. 2019; Hsue and Visser 2020; Leroux et al. 2020; Slack et al. 2012; Worsman et al. 2022), and only two of them evaluated the effect of the operator’s experience on the reliability of transthoracic echocardiography (Pedersen et al. 1996; Chetboul et al. 2003).

The present study aimed at evaluating the reproducibility and repeatability of echocardiographic parameters in dogs by analyzing measurements obtained from several veterinary cardiologists with different levels of experience and comparing them to the ones obtained from two board-certified operators, here considered as the “gold standard”. Furthermore, we tried to assess whether different formative paths have an influence on the variability of the echocardiographic measurements.

## Materials and methods

### Operators

A total of 51 operators with different experience in echocardiography have been included in this study. Two diplomates of the European College of Veterinary Internal Medicine (ECVIM) - Cardiology (CMB and RAS) were also included, and their echocardiographic examination of each dog has been used as gold standard (GS).

### Ultrasound machines

The echocardiographic examination was carried out by using two different ultrasound machines, one for each GS. The ultrasound machine used by the GS was also used by every operator who performed an echocardiographic examination on the same dog as the GS. In particular, CMB used Esaote MyLab Class C (Esaote; Genova, Italy) while RAS used Esaote MyLab 30 Gold (Esaote; Genova, Italy); both of them were equipped with phased array probes 2/2.5/3.5 MHz (Esaote; Genova, Italy).

### Animals

Ten dogs were enrolled for this study, 5 Golden Retrievers and 5 Cavalier King Charles Spaniels. Echocardiographic examinations were made in private clinics, during routinary clinical practice, and the owner’s written consent for each animal was obtained before enrollment. Echocardiography is considered a non-invasive procedure; therefore, this study has been conducted in respect of the European directive 2010/63/EU of the European Parliament and of the Council on the protection of animals used for scientific purposes. Echocardiographic examination was performed on each dog by one GS and several operators on the same day; this number differed from patient to patient and is summarized in Table 1. Each GS used an ultrasound machine (named A for CMB, and B for RAS) which was used also by the operators that performed the echocardiographic examinations along with the GS.

**Table 1:** Dogs divided according to the GS who performed the echocardiographic examination, and number of operators who evaluated them

	Dogs										
Gold standard	**1**	**2**	**3**	**4**	**5**	**6**	**7**	**8**	**9**	**10**	**Total**
CMB_A_	0	11	3	0	1	0	1	2	2	1	21
RAS_B_	17	0	0	7	0	6	0	0	0	0	30
Total	17	11	3	7	1	6	1	2	2	1	51

A = Esaote MyLab Class C, used by CMB and the operators; B = Esaote MyLab 30 Gold, used by RAS and the operators

### Echocardiographic examination

The animals were not sedated for the examination but were gently restrained in right and left lateral recumbence. An electrocardiogram was recorded simultaneously. Off-line analysis was made after acquiring images and recordings; at list 3 clips were obtained for each parameter. Two-dimensional echocardiographic parameters of the left ventricle (LV) were measured on the left apical 4-chamber view using the blood-tissue interface. After the selection of end-diastolic frames (at the beginning of the QRS complex, with the mitral valve closed) and end-systolic frames (last frame before the mitral valve opening), the LV area was measured by tracing the endocardial border; maximal LV length was obtained from the middle of a line that connects the hyperechoic areas of the mitral valve annulus to the endocardial border of the LV apex. LV end-diastolic and end-systolic volumes were estimated through the area-length method (Schiller et al. 1989). Aortic annulus diameter was measured from a right parasternal long-axis 5-chamber view, while the pulmonary artery annulus diameter was measured from a right parasternal short-axis view of the base of the heart. Both measurements were obtained through the inner-edge to inner-edge method in early systole at the hinge points of the valvar leaflets (Bussadori et al. 2001; Koplitz et al. 2006). The aortic root has been evaluated on the left parasternal left ventricular outflow view through the inner-edge to inner-edge measurements of the diameters at the sinuses of Valsalva and at the sinotubular junction; the ascending aorta diameter was then measured at a distance from the sinotubular junction equal to the diameter of the sinotubular junction itself. These three measurements were obtained at end-diastole (Pradelli et al. 2014). M-mode parameters were measured from a right parasternal short-axis view at the level of the papillary muscles by using the leading-edge to leading-edge method (Bonagura and Luis Fuentes 2000). Table 2 includes the list of the 15 echocardiographic parameters analyzed in the present study.

**Table 2:** List of the 15 echocardiographic parameters (measured or calculated) analyzed in the study

Echocardiographic parameter	Abbreviation
Aortic valve annulus diameter	AVA
Pulmonic valve annulus diameter	PVA
Aortic root at the sinuses of Valsalva	VLS
Aortic root at the sinotubular junction	STJ
Ascending aorta	AA
Left ventricle length in diastole	2D-LVL_d_
Left ventricle length in systole	2D-LVL_s_
Left ventricle volume in diastole	2D-LVV_d_
Left ventricle volume in systole	2D-LVV_s_
Left ventricular end-diastolic internal diameter	M-LVID_d_
Left ventricular end-systolic internal diameter	M-LVID_s_
Interventricular septal thickness in diastole	M-IVS_d_
Interventricular septal thickness in systole	M-IVS_s_
Left ventricular free wall thickness in diastole	M-LVW_d_
Left ventricular free wall thickness in systole	M-LVW_s_

### Statistical analysis

This is an observer agreement study. Considering inter-operator reliability as the reproducibility of measurements among operators, the coefficient of inter-operator reliability has been used to calculate the consistency of measurements and the extent at which the operators are interchangeable. It is defined as the covariance between two measurements made by different operators on the same patient, divided by the total variance. Considering intra-operator reliability as the reproducibility of repeated measurements by the same individual, the coefficient of intra-operator reliability has been used. It is regarded as estimates of the overall intra-operator reliability across all operators (Eliasziw et al. 1994). Using the Proc Mixed of SAS software (Base SAS® 9.4. SAS Institute Inc. Cary, NC), Inter-operator and Intra-operator Correlation Coefficients (CCs) were estimated for each parameter and their values were interpreted according to Koo and Li (2016): CCs lower than 0.50 are indicative of poor reliability, values between 0.50 and 0.75 indicate moderate reliability, values between 0.75 and 0.90 indicate good, and values greater than 0.90 indicate excellent reliability.

In order to assess the agreement between measurements from the GS and the operators, the Concordance Correlation Coefficient (CCC) was calculated (Lin 1989).

For each echocardiographic parameter, the deviation of the measure detected by the operator from the one detected by the GS (GS-O%) was calculated with the following formula and expressed as a percentage:

$$GS-O= \frac{GS measure-Operator measure}{GS measure}$$

The deviation of the measure from the GS indicates underestimation or overestimation by the operator if positive or negative, respectively. The absolute value of this measure (|GS-O|%) was also considered and was grouped into 11 classes in order to calculate the cumulative frequencies (0, 0.01–0.05, 0.06–0.10, 0.11–0.15, 0.16–0.20, 0.21–0.25, 0.26–0.30, 0.31–0.40, 0.41–0.50, 0.51–0.75, 0.76).

In a first step, “breed” and “ultrasound machine” were considered as confounding factors and therefore included in the statistical model as explanatory variables; however, since their effect was not significant for all the echocardiographic parameters, they were removed from the model.

For 39 out of 51 operators, information were collected relative to: (a) years of enrollment to the Italian society of veterinary cardiology (< 5 or > 5 years); (b) participation in cardiology congresses, seminars or courses in the last 5 years (< 10 or > 10 congresses); (c) years of echocardiography practice (< 5 or > 5 years); (d) time of professional activity dedicated to cardiology (< 50% or > 50%); (e) number of echocardiographic examinations per month (< 20 or > 20). The |GS-O|% variability was analyzed using a mixed model including the fixed effects of the abovementioned variables (a to d), the echocardiographic parameter, and the random effects of the dog and the operator. Least square means were separated by pair-wise t-test. Statistical differences were declared at a *P* value < 0.05.

## Results

### Demographics

Three of the ten dogs enrolled in the present study were females (1 entire and 2 neutered), and 7 were males (4 entire and 3 neutered); they were 1–7 years old. Golden Retriever weighted between 26.3 and 42.7 kg, while Cavalier King Charles Spaniels’ weight varied between 6.2 and 12.1 kg. All the dogs had to be clinically healthy at the time of the echocardiography based on clinical examination, blood analysis and serum chemistry.

### Inter-operator and intra-operator reliability

The values found for the intra-operator CC are clearly higher than those related to inter-operator CC variability, the range being between 0.71 and 0.97 (Table 3). The intra-operator reliability, with the exception of the M-LVW_s_ (0.73) and M-IVS_s_ (0.71), is good for 6 parameters (2D-LVL_s_, 2D-LVV_s_, M-LVID_s_, M-IVS_d_, M-LVID_d_ and M-LVW_d_) and excellent for the remaining 7 (AVA, PVA, AA, STJ, VLS, 2D-LVL_d_ and 2D-LVV_d_), with values between 0.75 and 0.85 and between 0.90 and 0.97, respectively.

**Table 3:** Intra- and inter-operator correlation coefficients for the echocardiographic measurements

Echocardiographic parameter	Inter-operator CC	Intra-operator CC
AVA	0.80	0.97
PVA	0.67	0.92
VLS	0.89	0.96
STJ	0.84	0.94
AA	0.79	0.95
2D-LVL_d_	0.71	0.92
2D-LVL_s_	0.60	0.84
2D-LVV_d_	0.63	0.90
2D-LVV_s_	0.11	0.79
M-LVID_d_	0.73	0.85
M-LVID_s_	0.76	0.81
M-IVS_d_	0.54	0.76
M-IVS_s_	0.38	0.71
M-LVW_d_	0.27	0.75
M-LVW_s_	0.44	0.73

CC = correlation coefficient

Poor reliability: < 0.50; moderate reliability: ≥ 0.50 and < 0.75; good reliability: ≥ 0.75 and < 0.90; excellent reliability: ≥ 0.90

The inter-operator reliability does not reach an excellent value for any parameter, and for four echocardiographic measurements it shows a poor CC, less than 0.5 (2D-LVV_s_, M-LVW_d_, M-LVW_s_ and M-IVS_s_). The level is moderate (0.54–0.73) for 6 parameters (PVA, 2D-LVL_d_, 2D-LVL_s_, 2D-LVV_d_, M-IVS_d_, and M-LVID_d_) and good for the remaining 5.

### Concordance with the gold standard

The concordance between GS and operators’ measurements is excellent only for VLS (0.92), and poor for 2D-LVV_s_ (0.47), M-IVS_d_ (0.41), and M-LVW_d_ (0.28). The CCCs for the other parameters are moderate to good and are summarized in Table 4.

**Table 4:** Concordance correlation coefficients for the echocardiographic measurements, and their class limits

Echocardiographic parameter	CCC	Lower CL	Upper CL
AVA	0.85	0.80	0.88
PVA	0.65	0.57	0.72
VLS	0.92	0.89	0.94
STJ	0.80	0.74	0.84
AA	0.57	0.47	0.66
2D-LVL_d_	0.72	0.64	0.78
2D-LVL_s_	0.62	0.53	0.70
2D-LVV_d_	0.54	0.45	0.63
2D-LVV_s_	0.47	0.34	0.58
M-LVID_d_	0.81	0.76	0.85
M-LVID_s_	0.78	0.72	0.82
M-IVS_d_	0.41	0.31	0.49
M-IVS_s_	0.52	0.42	0.61
M-LVW_d_	0.28	0.15	0.40
M-LVW_s_	0.64	0.56	0.71

CCC = concordance correlation coefficient; CL = class limit

Poor concordance: < 0.50; moderate concordance: ≥ 0.50 and < 0.75; good concordance: ≥ 0.75 and < 0.90; excellent concordance: ≥ 0.90

**Fig. 1:**
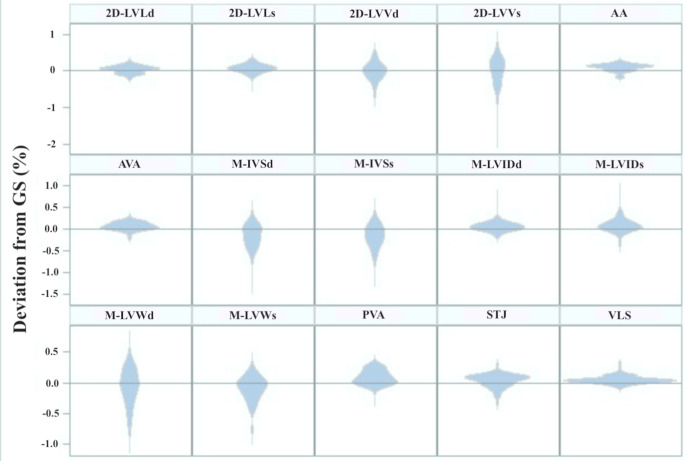
Violin plots that represent the distributions of the GS-O% variable for each echocardiographic measurement. On the y-axis the variable is represented: values above 0 represent the underestimation of the measure, while values under 0 represent the overestimation. On the x-axis the operators are represented: the larger the violin is, the higher the number of operators that have the same deviation from the GS is

Differences were found between echocardiographic parameters with respect to the distributions of the GS-O% variable (Fig. 1). The violin plots show that the operators tend to underestimate the measure of most of the parameters compared to the GS, the values being concentrated mostly above 0. For parameters M-LVW_d_, M-LVW_s_, M-IVS_d_, M-IVS_s_ there is a tendency to overestimate the measure. Furthermore, for 2D-LVV_s_, M-LVW_d_, M-LVW_s_, M-IVS_d_ and M-IVS_s_ there is a considerable variability of the deviations, while for the remaining parameters the difference between the GS and the operator measurements is limited.

**Fig. 2:**
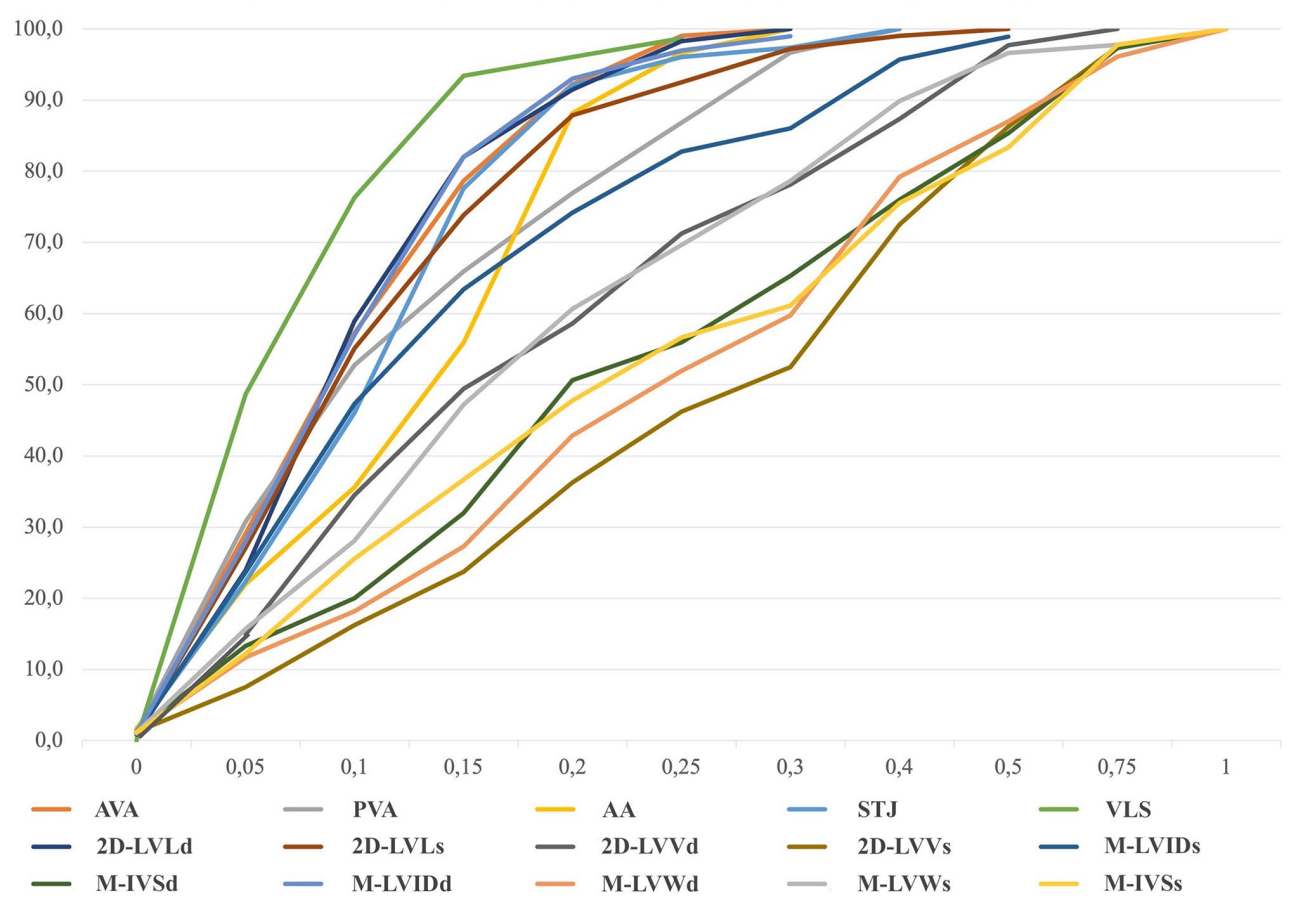
Cumulative curves of deviation from the GS measurements. On the y-axis the percentage of the operators is represented. On the x-axis deviation classes are represented. VLS appears to be the echocardiographic measurement that shows the lower deviation from the GS among the operators, while 2D-LVV_s_ is the parameter with the highest deviation

The cumulative curves of |GS-O|% variable show the smallest deviations between the GS and operator measures for the parameter VLS: in fact, 48.7%, 76.3% and 93.4% of operators show a deviation of 0.05, 0.10 and 0.15 from the GS, respectively. On the contrary, for the same deviation classes of 2D-LVVs, the frequency of operators is very low and equal to 7.5%, 16.3% and 23.8%, respectively (Fig. 2).

### Evaluation of the effect of formative experiences

Through the analysis of the answers gathered regarding each operator’s experience (Supplementary Material Tables S1 to S5), only the number of echocardiographic examinations performed by operator per month appears to significantly affect the variability of |GS-O|% (*P* = 0.039). The measurements taken by operators who perform more than 20 echocardiographic examinations per month show a significantly smaller difference from GS’ measurements (14.94 ± 0.94%) compared to operators who perform less than 20 echocardiographic examinations per month (17.55 ± 1.25%), as shown in Table 5.

**Table 5:** Least square means and standard errors relative to |GS-O|% for number of echocardiographic examinations per month

Source of variability	LSM	SE
10–20 echocardiographic examinations	17.55	1.25
> 20 echocardiographic examinations	14.94	0.94

LSM = least square mean; SE = standard error

## Discussion

In veterinary medicine, many studies focused on assessing the reliability of echocardiographic measurements in dogs. However, in most of these studies the echocardiographic examination was performed by highly trained or board-certified operators who then proceeded with the measurement of the echocardiographic parameters of interest (Dukes-McEwan et al. 2002; Abbott and Gentile-Solomon 2017; Hsue and Visser 2020). Differently, other studies engaged a board-certified operator for the execution of the echocardiographic examination and a few highly trained observers for the collection of measurements (Gentile-Solomon and Abbott 2016; Visser et al. 2019). Therefore, the present study is the first to engage a large number of operators from multiple institutions and with different levels of experience to assess the reliability and reproducibility of different 2D and M-mode echocardiographic parameters in dogs, as well as the concordance with two board-certified operators considered as the gold standard.

As already reported in literature, our results show that the intra-operator reliability is better than the inter-operator’s (Dukes-McEwan et al. 2002), with only M-IVS_s_ and M-LVW_s_ that show moderate intra-operator repeatability (M-IVS_s_ = 0.71, M-LVW_s_ = 0.73), while the other parameters vary from good to excellent. On the contrary, the inter-operator correlation coefficient does not reach excellent values for any echocardiographic parameter, and for the measurements that show poor reproducibility, such as M-IVS_s_ (0.38), M-LVW_s_ (0.44) and M-LVW_d_ (0.27), interchangeability between operators is not recommended. The concordance correlation coefficients with the GS confirm the poor reliability of these parameters and M-IVS_d_, and the violin plots, which graphically represent the GS-O% distribution, show how operators tend to overestimate their measurement when compared to the GS; M-IVS_s_, M-IVS_d_, M-LVW_s_, and M-LVW_d_ are the parameters with the higher deviation from the GS. These results demonstrate how some of the M-mode-obtained parameters are the least reliable ones. However, our results are in contrast with the study of Dukes McEwan et al. (2002), which reported the lowest coefficients of variation for M-mode measurements (Dukes-McEwan et al. 2002). The Authors hypothesize that this difference could be due to the different experience of the operators of this study in comparison with the Dukes McEwan’s, where only two experienced echocardiographers were involved (Dukes-McEwan et al. 2002). In fact, measurements of the interventricular septum and the left ventricular free wall in systole and in diastole are greatly influenced by the alignment of the cursor, the blood-tissue interface, the myocardial contractility, the presence of the papillary muscles and the *cordae tendinae*, as well as by the interference of the right ventricle for the interventricular septum and the hyperechoic area of the pericardium for the left ventricular free wall. Results presented in this study are in line with what is reported for the cat by Chetboul et al. (2003), in a study in which operators with different experience were involved (Chetboul et al. 2003). In fact, results obtained by Chetboul et al. in cats reported the highest coefficients of variation for M-IVS_d_, M-IVS_s_ and M-LVW_s_ for the operator with poor experience in echocardiography (< 4 months of training); interestingly, also the most experienced board-certified operator reported high coefficients of variation for M-IVS_s_ and M-LVW_d_ when compared to the other parameters (Chetboul et al. 2003). Lastly, the study showed a tendency for the less experienced echocardiographers to overestimate measurements (Chetboul et al. 2003), accordingly to what we reported for M-LVW_d_, M-LVW_s_, M-IVS_d_, M-IVS_s_, which are the echocardiographic parameters with the highest deviation from the GS. Considering these results, authors suggest that a good optimization of the image must be the primary lesson of every echocardiography course, since a good quality image is pivotal for the acquisition of reliable measurements; furthermore, it is imperative to acquire more than one video or image for every view in order to choose the best available frame for the measure. Lastly, for the M-mode, the recommendation is to collect left ventricular parameters using and end-expiratory frame, in order to minimize the influence of ventilation on the right heart.

In contrast with measurements of the interventricular septum and the left ventricular free wall, the internal diameters of the left ventricle in systole and diastole show moderate to good inter-operator reproducibility (M-LVID_d_ = 0.73, M-LVID_s_ = 0.76) and good concordance with the GS (M-LVID_d_ = 0.81, M-LVID_s_ = 0.78). Furthermore, M-LVID_d_ shows a small deviation between the GS and the operators’ measurements through its cumulative curve (28% and 82% of operators show a deviation of 0.05 and 0.15 from the GS, respectively). The violin plots of M-LVID_d_ and M-LVID_s_ show that operators tend to underestimate them, but the deviation from the GS’ measurement is low. The reliability of these parameters is fundamental since they allow the operator to assess important aspects of the left ventricle, such as its systolic function and its degree of dilation. These results agree with what is reported by Dukes-McEwan et al. (2002) who confirm the lowest coefficient of variation for M-LVID_d_ (7.74%) among all the analyzed M-mode parameters; a low coefficient of variation was also found for M-LVID_s_ (10.43%), so the authors suggest that for these parameters a change of over 10% is likely to be significant (Dukes-McEwan et al. 2002).

Results show a poor inter-operator correlation coefficient for 2D-LVV_s_ (0.11), and yet the intra-operator correlation coefficient is good for this parameter (0.79); this could indicate that the operators tend to repeat the same error in the measurement of 2D-LVV_s_. The concordance correlation coefficient that confronts the operators with the GS confirms the poor reliability of this parameter (0.47), and its cumulative curve shows the highest deviation from the GS when compared to all other parameters (7.5% and 23.8% of operators show a deviation of 0.05 and 0.15 from the GS, respectively). The violin plot shows that operators tend to overestimate 2D-LVV_s_ and confirms the high deviation from the GS. These results agree with the study of Dukes-McEwan et al. (2002), which reports for 2D-LVV_s_ a moderate to high coefficient of variation (24.14%), the highest value obtained among the other 2D-obtained parameters of the left ventricle (Dukes-McEwan et al. 2002). A more recent study reported a high inter-observer intraclass correlation coefficient for this parameter; however, according to the scheme of that study, images were obtained only by one operator and measurements were then made by 3 expert operators (Visser et al. 2019). Errors in the measurement of 2D-LVV_s_ made by various operators could be due to the blood-tissue interface, which is more difficult to detect in systole than in diastole because of the presence of the papillary muscles that can be considered differently among operators when tracing the endocardial board. Attention must be paid in theoretical and practical courses in teaching how to correctly measure this parameter since an overestimation in its detecting could lead to the misinterpretation of the systolic function of the left ventricle obtained in 2D echocardiography. Moderate reproducibility is demonstrated for the inter-operators’ measurements of 2D-LVL_s_ (0.60), 2D-LVL_d_ (0.71), and 2D-LVV_d_ (0.63), instead. The concordance correlation coefficients confirm a moderate agreement for these 2D-obtained parameters when compared to the GS.

The inter-operator correlation coefficient does not reach excellent values for any parameter, but show a good reproducibility of different measurements, such as the aortic annulus (0.80), and the aortic root measurements obtained on the left parasternal left ventricular outflow view (VLS = 0.89, STJ = 0.84, AA = 0.79). A moderate inter-operator correlation coefficient is reported for the pulmonic valve annulus (0.67). Although aortic root measurements (VLS, STJ and AA) have good inter-operator correlation coefficients, through the analysis of the concordance with the GS, only VLS reaches an excellent value (0.92), while STJ confirms a good agreement with the GS (0.80), and AA only reaches a moderate value (0.57). Measurement of the pulmonic valve annulus confirms a moderate concordance with the GS (0.65), and the aortic annulus remains a reliable measurement with a good concordance correlation coefficient with the GS (0.85). Cumulative curves confirm that VLS is the parameter that shows the smallest deviation between the GS and the operators’ measurements (48.7% and 93.4% of operators show a deviation of 0.05 and 0.15 from the GS, respectively), while violin plots show that for AVA, PVA, VLS, STJ and AA, operators tend to underestimate the measurements. Basing on these results, measurement of the aortic annulus is interchangeable between operators, and this may be because it is measured in early systole, while the valve is open, so the blood-tissue interface is clear. On the contrary, AA is more difficult to be correctly obtained, because it is a derived measurement since it is acquired at a distance from the sinotubular junction equal to the diameter of the sinotubular junction itself. The reliability of the aortic annulus and root measurements demonstrated in this study is very important since measurements and evaluations of the aorta are pivotal for the diagnosis of congenital heart diseases. The moderate values obtained for the pulmonic valve, on the contrary, suggest that a higher attention should be paid in teaching how to obtain this parameter, since interchangeability in its measurement cannot be confirmed yet.

Lastly, the analysis of the answers gathered regarding the operators’ experience shows that only the number of echocardiographic examinations performed per month significantly reduces the variability with the GS. Therefore, a specialist clinical activity, more than the acquired theoretical knowledge, affects the reproducibility of the echocardiographic examination.

This study presents some limitations. Firstly, data have been acquired during practical courses held by the GSs, so the operators could have been influenced by what they had learnt during the course. This may have induced a sort of standardization, otherwise absent, among the operators. Secondly, not all the echocardiographic measurements that are normally used in clinical practice have been included in this study, so it might be interesting to evaluate these other measurements in the future using the same scheme hereby proposed. Lastly, two different ultrasound machines have been used and different dogs have been engaged in the study, and this could have been responsible for the great differences between the inter-operator correlation coefficients and the concordance correlation coefficients, along with the different experience of the involved operators. However, the fixed effects of the variables “dog” and “operator” have been included in all the analyses, so they do not have to be considered; furthermore, the different experience of the operators must be regarded as a strength point because it reflects a real population of veterinary echocardiographers.

In conclusion, this is the first study to assess the reproducibility and repeatability of numerous echocardiographic parameters through the analysis of data acquired from a large number of operators with different levels of experience and the comparison with two board-certified operators. Furthermore, this is the first time that the effect of the operators’ experience, both theoretical and practical, on the reliability of the echocardiographic examination has been assessed. Results show that M-IVS_s_, M-IVS_d_, M-LVW_s_, M-LVW_d_, and 2D-LVV_s_ are the least reliable parameters since they show a high deviation from the GS and/or a poor inter-operator correlation coefficient. Attention must be paid in theoretical and practical courses in teaching how to correctly measure these parameters. On the contrary, M-LVID_d_, M-LVID_s_, AVA, VLS, and STJ are the most reliable echocardiographic measurements, demonstrating that all the operators are able to correctly assess left ventricular systolic function and dilation, as well as to precisely evaluate the aortic annulus and root, fundamental aspects for the diagnosis of congenital and acquired heart diseases. Furthermore, a specialist clinical activity affects the reliability of the echocardiographic examination.

### Electronic supplementary material

Below is the link to the electronic supplementary material.

Supplementary Material 1
