# Supplementary material for: A study of the inter- and intra-operator variability on selected echocardiographic measurements in dogs
Source: Vet Res Commun. 2023 Jun 23;47(4):2323–31. doi: 10.1007/s11259-023-10154-6 (PMC10698089; doi:10.1007/s11259-023-10154-6)
Supplement: Supplementary file 1 — Supplementary Material 1 [file 11259_2023_10154_MOESM1_ESM.docx]

**Table S1** Frequency of operators and echocardiographic measurements used in the analysis of the answers gathered about the years of enrollment to the Italian society of veterinary cardiology

| Years of enrollment to the Italian society of veterinary cardiology | Operators | Echocardiographic measurements |
| --- | --- | --- |
| < 5 | 8 | 337 |
| > 5 | 23 | 901 |
| No | 8 | 307 |

**Table S2** Frequency of operators and echocardiographic measurements used in the analysis of the answers gathered about the participation in cardiology congresses, seminars or courses in the previous 5 years

| Number of congresses, seminars, or courses in the previous 5 years | Operators | Echocardiographic measurements |
| --- | --- | --- |
| < 10 | 14 | 575 |
| > 10 | 25 | 970 |

**Table S3** Frequency of operators and echocardiographic measurements used in the analysis of the answers gathered about the years of echocardiography practice

| Years of echocardiography practice | Operators | Echocardiographic measurements |
| --- | --- | --- |
| < 5 | 3 | 126 |
| > 5 | 36 | 1419 |

**Table S4** Frequency of operators and echocardiographic measurements used in the analysis of the answers gathered about the time of professional activity dedicated to cardiology

| Time of professional activity dedicated to cardiology (%) | Operators | Echocardiographic measurements |
| --- | --- | --- |
| < 50 | 18 | 740 |
| > 50 | 21 | 805 |

**Table S5** Frequency of operators and echocardiographic measurements used in the analysis of the answers gathered about the number of echocardiographic examinations per month

| Number of echocardiographic examinations per month | Operators | Echocardiographic measurements |
| --- | --- | --- |
| < 20 | 12 | 491 |
| > 20 | 27 | 1054 |
